# Enhanced cognitive demodulation with artificial intelligence

**DOI:** 10.1038/s41598-020-77262-0

**Published:** 2020-11-20

**Authors:** Hang Ren, Sang-Hee Shin, Stepan Lucyszyn

**Affiliations:** Department of Electrical and Electronic Engineering, Imperial College London, London, SW7 2AZ UK

**Keywords:** Lasers, LEDs and light sources, Electrical and electronic engineering

## Abstract

The low-cost ‘THz Torch’ wireless link technology is still in its infancy. Until very recently, inherent limitations with available hardware has resulted in a modest operational figure of merit performance (Range $\times$ Bit Rate). However, a breakthrough was reported here by the authors, with the introduction of ‘Cognitive Demodulation’. This bypassed the thermal time constant constraints normally associated with both the thermal emitter and sensor; allowing step-change increases in both Range and Bit Rate with direct electronic modulation. This paper concentrates on advancements to the bit error rate (BER) performance. Here, separate techniques are introduced to the demodulation software that, when combined, result in enhanced Cognitive Demodulation. A factor of more than 100 improvement in BER was demonstrated within the laboratory and approximately a 60-fold improvement in a non-laboratory environment; both at the maximum Range and Bit Rate of 2 m and 125 bps, respectively, demonstrated recently. Moreover, demodulation speed is increased by almost a factor of 10,000; allowing for real-time demodulation while easing future computational hardware requirements. In addition to these software advancements, the paper demonstrates important improvements in hardware that has brought the technology out of the laboratory, with field trials being performed within an office corridor.

## Introduction

The first thermal infrared wireless digital communications system, known as the ‘THz Torch’, was introduced in 2011^1^. The philosophy behind this technology is to implement a secure and robust communications channel, while being extremely low cost-making it affordable for future ubiquitous applications. Here, the thermal-based physical layer hardware consists of a transmitter (employing miniature incandescent light bulbs or thermal emitters) and receiver (employing pyroelectric infrared (PIR) motion sensors) that supports wireless data transfer via a modulated noise carrier. The same group then went on to investigate various aspects associated with the ‘THz Torch’ technology, from systems level architectures down to device level enabling technologies, while trying to maintain the low-cost philosophy^2–8^. From this, two major scientific breakthroughs were published^7,8^: the first was in 2014, which demonstrated the secure nature of this technology^7^; while in early 2020, the thermal time constants associated with the hardware were bypassed by introducing the new concept of ‘Cognitive Demodulation’^8^. This powerful technique has wide-ranging implications for future communications (underwater, terrestrial and aerospace) and sensor technologies (sonar/radar/lidar and imaging), making them more resilient when operating in harsh environments. For example, the concept of having an adaptive, time-variant matched filter can be applied to future generations (+5G) of cellular/mobile communications systems, helping to maintain bit-error rate levels as physical channels degrade. The concept was applied to this extreme case, employing a thermal noise-based carrier system, but can be adapted to conventional sinusoidal-based carrier systems. Basic Cognitive Demodulation relies on the complete system being modelled, with the level of success being dependent on the accuracy of the individual parameter values. This paper first introduces a framework for timing synchronization and demodulation. Then an emitter calibration method is presented to complete the model of the entire system. This complete system model is combined with advanced signal processing to improve the timing synchronization and then artificial intelligence is introduced to enhance Cognitive Demodulation at the receiver. With the latter, recent advances in machine learning^9,10^ (from the computer science community) provides new perspectives and tools for communications applications. As an example, new demodulation algorithms can be designed by adapting powerful function approximators (e.g., neural network) to different modulation schemes, converting measured analogue waveforms into digital symbols^11–15^. Using the ‘THz Torch’ wireless link as a harsh environment testbed, this paper will introduce the following three techniques for enhancing Cognitive Demodulation: Emitter Calibration; Timing Synchronization; and Neural Networks.

## Results

The architecture for this ‘THz Torch’ wireless communications link has been described at length in our recent paper^8^ and will not be repeated here for brevity.

### Non-laboratory experimental setup

**Figure 1:**
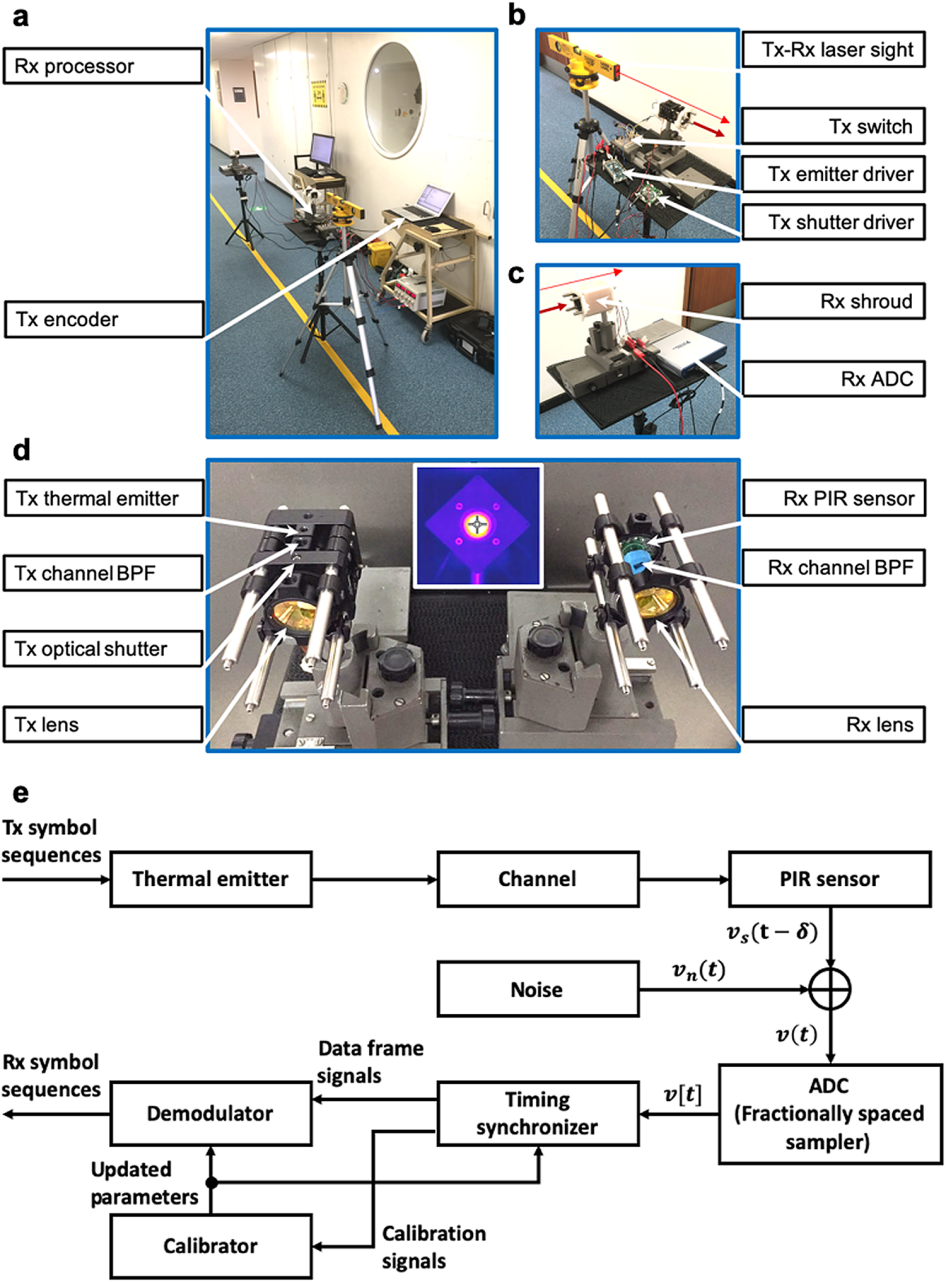
(**a–d**) Practical field trials for a 2 m ‘THz Torch’ wireless link. (**a**) The complete ‘THz Torch’ link showing the transmitter (Tx) and receiver (Rx). (**b**) Zoomed-in view of the transmitter. (**c**) Zoomed-in view of the receiver. (**d**) Zoomed-in view of the transmitter and receiver front-end hardware. The insert in (**d**) shows the thermal image of the transmitter’s front-end hardware with the thermal emitter in the ON-state (the cross-hairs from the camera image indicates the centre of the Tx lens). (**e**) Basic block diagram for the ‘THz Torch’ transceiver architecture.

The recently reported 0.5–2 m ‘THz Torch’ wireless links were set up within a laboratory environment^8^, with the use of an optical bench and precision alignment rails. Here, measurements are now performed in a non-laboratory setting (within an office corridor, as shown in Fig. 1a–d), with reference experiments also performed within the laboratory. Instead of using precision alignment rails, the field trials employed simple laser alignment, with the use of two 3D-printed guide holes in both the transmitter (Tx) and receiver (Rx) setups. In addition, a 3D-printed shroud was used by the receiver to suppress microphonic noise and ambient temperature fluctuations.

### Receiver architecture and framework

Figure 1e shows the basic block diagram for the ‘THz Torch’ transceiver architecture. The up-converter and down-converter, which are commonly seen in conventional digital communications links, are implicitly included in the thermal emitter and PIR sensor blocks, respectively. The analog-to-digital converter (ADC) is exploited as a fractionally spaced sampler, with sampling rate chosen to be an integer multiple of the symbol rate. The timing synchronizer locates the temporal boundary for each predefined training sequence. The demodulator then processes the data frame signals to generate the received symbol sequence. The calibrator implements the thermal emitter (discussed later) and PIR sensor^8^ calibrations; updating their system parameters from synchronized calibration signals. The output from the calibrator is sent to the demodulator and the timing synchronizer; improving their future decision-making processes.

The received discrete baseband signal in Fig. 1e can be expressed as:

1 $$\begin{aligned} v[t] = v_s[t - \delta ] + v_n[t] \end{aligned}$$

where $v_s[t - \delta ]$ is a delayed noiseless received signal corresponding to the transmitted symbols; $\delta$ represents the time delay; and $v_n[t]$ is the sampled noise. Although the noise mechanism is complicated within our system, a reasonable approximation is that $v_n[t]$ is sampled white Gaussian noise (WGN) with two-sided power spectral density (PSD) $\sigma _n^2$. Equivalently, $v_n[t]$ are independent and identical distributed (I.I.D.) samples from zero-mean Gaussian distribution $\mathscr {N}(0, \sigma _n^2)$.

Timing synchronization aims at estimating the time delay $\delta$. It is achieved by matching the received signal to the expected noiseless signal corresponding to a sequence of predefined training symbols. The received signal for synchronization can be written as:

2 $$\begin{aligned} v[t] = v_{train}[t - \delta ] + v_n[t] \end{aligned}$$

where $v_{train}[t]$ is the expected noiseless signal corresponding to the sequence of training symbols. Timing synchronization can be formulated under the hypothesis testing framework^16^. Given two hypotheses:

3 $$\begin{aligned} \begin{aligned} H_{train}&: v[t] = v_{train}[t-\delta ] + v_n[t] \\ H_n&: v[t] = v_n[t] \end{aligned} \end{aligned}$$

where $H_{train}$ is the hypothesis corresponding to the signal model in Eq. (2) and $H_n$ is a noise-only dummy hypothesis; the objective of timing synchronization is to maximize a likelihood ratio w.r.t. $\delta$:

4 $$\begin{aligned} \delta ^{*} = arg\max _\delta \ \frac{p_\delta (v|H_{train})}{p(v|H_n)} = arg\max _\delta \ ln \ \frac{p_\delta (v|H_{train})}{p(v|H_n)} \end{aligned}$$

Once the timing synchronization is established, $\delta$ can be removed from Eq. (1) by simply shifting the expected noiseless signal in time. Given two possible symbol values $\{S0, \ S1\}$, with ON-OFF keying (OOK) modulation, there are three predefined training sequences for timing synchronization in our ‘THz Torch’ wireless link: the PIR sensor calibration sequence; the thermal emitter calibration sequence, which includes four leading *S*1 followed by twenty *S*0, sent at 50 Bd; and the fixed preamble $\{S1, S1, S1, S0\}$ for each data frame. The expected noiseless signal for the PIR sensor calibration sequence is essentially a step response^8^. The expected noiseless signal for the thermal emitter calibration sequence and the data frame preamble can be determined by solving Eq. (4)^8^, then Eq. (16)^8^ and finally Eq. (14)^8^ in our most recent previously reported work^8^. In Eq. (4)^8^, $W_e$ is switched between $W_R$ and 0, according to the training sequence. Initial conditions are $T(0)=T_0$ for Eq. (4)^8^ and $v'(0)=v(0)=0$ for Eq. (14)^8^.

The demodulator is exploited to retrieve the transmitted symbol sequence from each synchronized data signal. Assuming a transmitted symbol sequence $\{s_1, s_2, \ ... \ s_N\}$ with values $\{S0, \ S1\}$, the overall received signal is:

5 $$\begin{aligned} v[t] = \sum _{i=1}^N v_{s_i}^{s_{1:(i-1)}}[t - (i-1)\times T_S] + v_n[t] \end{aligned}$$

where $T_S$ is the samples per symbol interval and $v_{s_i}^{s_{1:(i-1)}}[t]$ is the expected noiseless signal of $s_i$ conditioned on all previous symbols $s_{1:(i-1)}$. Given our two symbol values $\{S0, \ S1\}$, there are two hypotheses for *v*[*t*] of $s_i$:

6 $$\begin{aligned} \begin{aligned} H_{S0}&: v[t] = v_{S0}^{s_{1:(i-1)}}[t] + v_n[t] \\ H_{S1}&: v[t] = v_{S1}^{s_{1:(i-1)}}[t] + v_n[t] \end{aligned} \end{aligned}$$

A demodulator that exploits the maximum likelihood (MLE) decision rule is represented by:

7 $$\begin{aligned} s_i^{*} = arg\max _{s_i} \ p(v|H_{s_i}) = arg\max _{s_i} \ ln \ p(v|H_{s_i}) \end{aligned}$$

The decision rule in Eq. (7) is applied iteratively to demodulate all the symbols in chronological order. Again, two possible noiseless received signals $v_{S0}^{s_1:(i-1)}[t]$ and $v_{S1}^{s_1:(i-1)}[t]$ can be obtained using Eq. (4)^8^, Eq. (16)^8^ and Eq. (14)^8^. Similarly, we have $W_e=0$ for $v_{S0}^{s_1:(i-1)}[t]$ and $W_e=W_R$ for $v_{S1}^{s_1:(i-1)}[t]$, with our chosen OOK modulation scheme. Here, the initial conditions *T*(0), *v*(0) and $v'(0)$ are decided by all previous symbols $s_{1:(i-1)}$ within the same frame. Without knowing *a prior* the true transmitted $s_{1:(i-1)}$, the demodulated symbols for $s_{1:(i-1)}$ are used instead.

This hypothesis testing framework for synchronization and demodulation is commonly used in digital transceiver design. However, our concept of a cognitive receiver can be justified for the following two reasons:

- Conventional digital systems rely on linear memoryless modulation. In contrast, with our system, we have a complex memory and nonlinear modulation in which the expected noiseless signal is defined by the current symbol and all previous symbols within the same frame. The $v_{s_i}^{s_{1:(i-1)}}[t]$ term in Eq. (5) and Eq. (6) shows this dependency by its superscript. Our solution constantly tracks the thermodynamics of the system to produce all possible hypotheses in Eq. (6).
- All expected noiseless signals used for synchronization and demodulation are implicitly dependent on the thermal emitter and PIR sensor parameters. Our solution adaptively calibrates these parameters using optimization approaches.

Following the framework developed here, four algorithms are introduced in this work to further enhance our cognitive ‘THz Torch’ receiver.

### Naive template matching and matched filter

The basic cognitive demodulation introduced in our previous work^8^ is intuitive. For synchronization, the following optimization problem is solved:

8 $$\begin{aligned} \delta ^*= arg\min _\delta \ \frac{1}{T_H} \sum _{t=0}^{T_H-1} (v[t] - v_{train}[t - \delta ])^2 \end{aligned}$$

where $T_H$ is a integer that represents a sufficiently large time horizon so that the energy of $v_{train}[t-\delta ]$ does not depend on $\delta$. An intuitive explanation for Eq. (8) is that the optimal time delay for timing synchronization minimizes the mean-square-error between the received signal and the delayed noiseless signal. Its counterpart for demodulation is given by:

9 $$\begin{aligned} s_i ^*= arg\min _{s_i} \ \frac{1}{T_S} \sum _{t=0}^{T_S-1} (v[t] - v_{s_i}^{s_{1:(i-1)}}[t])^2 \end{aligned}$$

where $T_S$ is a integer that represents the number of samples per symbol. Equation (9) finds the optimal $i-$th symbol $s_i ^*$, which minimizes the mean-square-error between the received signal *v*[*t*] and the expected noiseless signal corresponding to $s_i$. By deriving Eq. (8) and Eq. (9) from Eq. (4) and Eq. (7), respectively, we can unify the basic cognitive demodulation under our hypothesis testing framework; refer to here as naive template matching. Assuming $v_{train}[t_1]$ and $v_{train}[t_2]$ are independent for $\forall \ t_1, t_2 \in [0, T_H)$, Eq. (4) can be written as:

10 $$\begin{aligned} \begin{aligned} \delta ^{*}&= arg\max _\delta \ ln \ \frac{p_\delta (v|H_{train})}{p(v|H_n)} \\&= arg\max _\delta \ \sum _{t=0}^{T_H-1} ln \ \mathscr {N}(v[t]|v_{train}[t-\delta ], \sigma _n^2) - \sum _{t=0}^{T_H-1} ln \ \mathscr {N}(v[t]|0, \sigma _n^2) \\&= arg\max _\delta \ \sum _{t=0}^{T_H-1} ln \ \mathscr {N}(v[t]|v_{train}[t-\delta ], \sigma _n^2) \\&= arg\max _\delta \ \sum _{t=0}^{T_H-1} \ ln \ \Bigg\{\frac{1}{\sqrt{2 \pi \sigma _n^2}} exp \ \left[ -\frac{(v[t]-v_{train}[t-\delta ])^2}{2\sigma _n^2}\right] \Bigg\}\\&= arg\min _\delta \ \frac{1}{T_H} \sum _{t=0}^{T_H-1} (v[t] - v_{train}[t - \delta ])^2 \end{aligned} \end{aligned}$$

Following the same independent assumption, for Eq. (7), we have:

11 $$\begin{aligned} \begin{aligned} s_i^{*}&= arg\max _{s_i} \ ln \ p(v|H_{s_i}) \\&= arg\max _{s_i} \sum _{t=0}^{T_S-1} ln \ \mathscr {N}(v[t]|v_{s_i}^{s_{1:(i-1)}}[t], \sigma _n^2) \\&= arg\max _{s_i} \ \sum _{t=0}^{T_S-1} \ ln \ \Bigg \{\frac{1}{\sqrt{2 \pi \sigma _n^2}} exp \ \left[ -\frac{(v[t]-v_{s_i}^{s_{1:(i-1)}}[t])^2}{2\sigma _n^2}\right] \Bigg \} \\&= arg\min _{s_i} \ \frac{1}{T_S} \sum _{t=0}^{T_S-1} (v[t] - v_{s_i}^{s_{1:(i-1)}}[t])^2 \end{aligned} \end{aligned}$$

Derivations for this naive template matching assume that any two timestamps in the expected noiseless signal are independent. This assumption does not consider the fact that there are only a few possible noiseless signals expected and, thus, timestamps in one expected signal are closely dependent on each other. To overcome this shortcoming, we adapt the signal space and matched filter concepts used in conventional digital communications to our ‘THz Torch’ system. Here, matched filter based synchronization and demodulation are expressed by Eq. (12) and Eq. (13), respectively.

12 $$\begin{aligned} \delta ^*= arg\max _\delta \ (v *\mathscr {M}_{train})[\delta ] = arg\max _\delta \ \langle v[t], v_{train}[t-\delta ] \rangle \end{aligned}$$

where $\mathscr {M}_{train}[t] = v_{train}[-t]$ is the matched filter for $v_{train}[t]$; $(*)$ is the convolution operator; and $\langle , \rangle$ is the inner product operator.

13 $$\begin{aligned} \begin{aligned} s_i^*&= arg\max _{s_i} \ (v *\mathscr {M}_{s_i}^{s_{1:(i-1)}})[0] - \frac{\langle v_{s_i}^{s_{1:(i-1)}}[t], v_{s_i}^{s_{1:(i-1)}}[t]\rangle }{2} \\&= arg\max _{s_i} \ \langle v[t], v_{s_i}^{s_{1:(i-1)}}[t]\rangle - \frac{ \Vert v_{s_i}^{s_{1:(i-1)}}\Vert ^2}{2} \end{aligned} \end{aligned}$$

where $\Vert \cdot \Vert ^2$ represents the energy of a signal. Unlike conventional matched filters, $\mathscr {M}_{s_i}^{s_{1:(i-1)}}[t]$ in Eq. (13) now varies with the previously transmitted symbols. Furthermore, all matched filters can perceive the system variation by employing calibrated emitter and sensor parameters.

The matched filter algorithm is essentially an extension of naive template matching to signal space. Since there are only a few possible noiseless signals expected, a low dimensional signal space can be constructed and each hypothesis in Eq. (3) and Eq. (6) can be projected to a Gaussian distribution in signal space. Conditional likelihoods in Eqs. (4) and (7) are simplified to probabilities under these projected Gaussian distributions. Any noise signal component, which is orthogonal to the signal space, has no contribution to the result and can, thus, be dropped. In addition, the matched filter remains optimal when there is a scaling factor between the predicted and the real noiseless signal expected.

**Figure 2:**
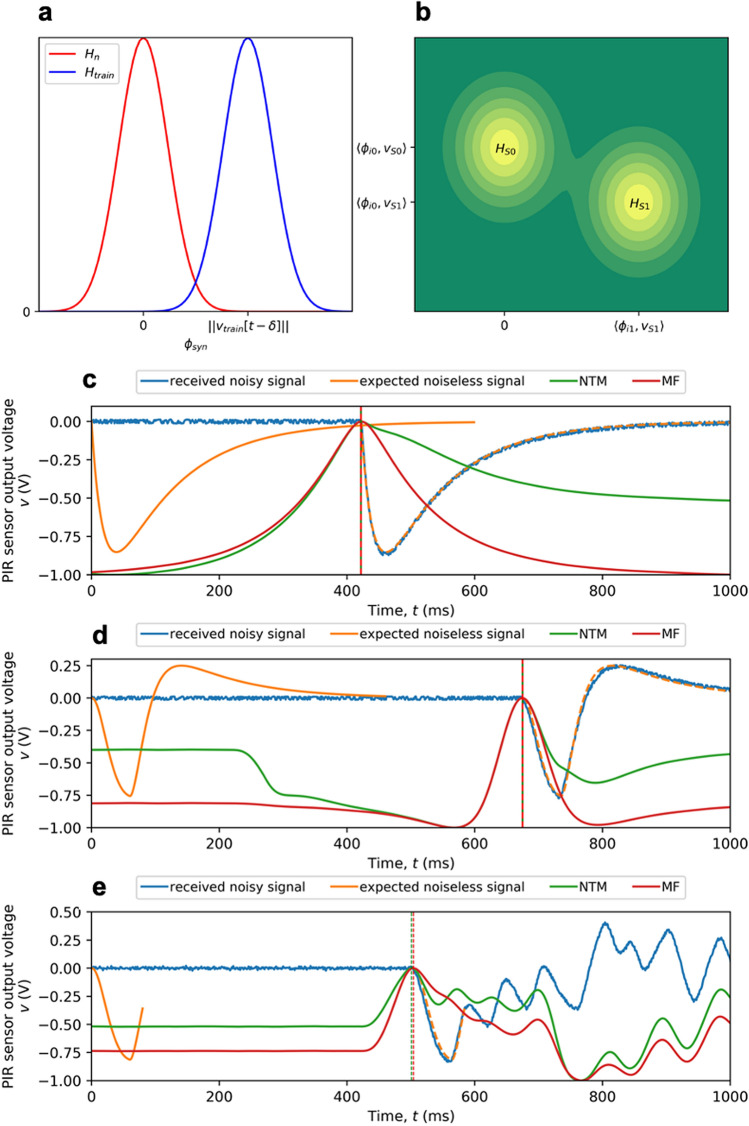
(**a–b**) Matched filter hypothesis in signal space. **(a)** One dimensional signal space for synchronization. **(b)** Two dimensional signal space (varying from symbol to symbol) for demodulation. (**c–e**) Timing synchronization for predefined training sequences demonstrated for the 2 m single-channel ‘THz Torch’ wireless link field trial: The naive template matching (NTM) objective in Eq. (8) and the matched filter (MF) objective in Eq. (12) are scaled and shifted to $[-1, 0]$ for visualization purposes. Each time delay estimation, indicated by a vertical dashed line, is obtained by locating the peak of its corresponding objective function. (**c**) Timing synchronization for PIR sensor calibration. (**d**) Timing synchronization for thermal emitter calibration. (**e**) Timing synchronization for data frame.

Figure 2a,b illustrates the matched filter operation in signal space. For synchronization, the signal space is one-dimensional with a basis $\phi _{syn}[t] = \frac{v_{train}[t-\delta ]}{\Vert v_{train}[t-\delta ]\Vert }$. Two hypotheses $H_{train}$ and $H_n$ are projected to the $\mathscr {N}(\Vert v_{train}[t-\delta ]\Vert , \sigma _n^2)$ and $\mathscr {N}(0, \sigma _n^2)$ distributions, respectively. The projection of the received signal *v*[*t*] onto the signal space is $\langle \phi _{syn}[t], v[t]\rangle$. Equation (12) is equivalent to maximizing the likelihood ratio w.r.t. the time delay $\delta$:

14 $$\begin{aligned} ln \ \frac{p_\delta (v|H_{train})}{p(v|H_n)} = ln \ \frac{\mathscr {N}(\langle \phi _{syn}[t], v[t]\rangle |\Vert v_{train}[t-\delta ]\Vert , \sigma _n^2)}{\mathscr {N}(\langle \phi _{syn}[t], v[t]\rangle |0, \sigma _n^2)} \end{aligned}$$

For demodulating the $i-$th symbol, the signal space is two-dimensional with the basis $\phi _{i0}[t]$ and $\phi _{i1}[t]$, calculated using Gramm-Schmidt orthogonalization:

15 $$\begin{aligned} \begin{aligned} \phi _{i0}[t]&= \frac{v_{S0}^{s_{1:(i-1)}}[t]}{\Vert v_{S0}^{s_{1:(i-1)}}[t]\Vert }\\ \phi _{i1}'[t]&= v_{S1}^{s_{1:(i-1)}}[t] - \langle v_{S1}^{s_{1:(i-1)}}[t], \phi _{i0}[t]\rangle \cdot \phi _{i0}[t]\\ \phi _{i1}[t]&= \frac{\phi _{i1}'[t]}{\Vert \phi _{i1}'[t]\Vert } \end{aligned} \end{aligned}$$

Hypotheses $H_{S0}$ and $H_{S1}$ in Eq. (6) are mapped to two Gaussian distributions:

16 $$\begin{aligned} \begin{aligned} H_{S0}: \ \mathscr {N}\bigg (\begin{bmatrix}\langle \phi _{i0}[t], v_{S0}^{s_{1:(i-1)}}[t] \rangle \\ 0\end{bmatrix}, \ \sigma _n^2 I\bigg ) \\ H_{S1}: \ \mathscr {N}\bigg (\begin{bmatrix}\langle \phi _{i0}[t], v_{S1}^{s_{1:(i-1)}}[t] \rangle \\ \langle \phi _{i1}[t], v_{S1}^{s_{1:(i-1)}}[t] \rangle \end{bmatrix}, \ \sigma _n^2 I\bigg ) \end{aligned} \end{aligned}$$

The projection of the received signal *v*[*t*] onto the signal space is given by:

17 $$\begin{aligned} \mathbf {proj}(v[t]) = \begin{bmatrix}\langle \phi _{i0}[t], v[t] \rangle \\ \langle \phi _{i1}[t], v[t] \rangle \end{bmatrix} \end{aligned}$$

The decision rule in Eq. (13) essentially chooses the hypothesis that assigns the largest probability to the received signal projection.

Figure 2c,d,e illustrates the synchronization achieved by naive template matching and the matched filter. Compared with the former, the latter’s objective in Eq. (12) tends to have a more steep peak at the optimal time delay. These results are consistent with Fig. 2c&d and deviate by 2 ms for data frame synchronization in Fig. 2e.

### Introduction of thermal emitter calibration

This section introduces a thermal emitter calibration algorithm to complete the model of the entire system. Obeying the principle of conservation of energy, while ignoring thermal convection, the following differential equation has been formulated^8^ to describe the thermodynamics of the emitter:

18 $$\begin{aligned} W_e(t) = C_e\frac{d T(t)}{d t} + k_e[T(t)-T_0] + \sigma _e\epsilon A_e T^{4}(t) \end{aligned}$$

where, $W_e\left( t\right)$ is the thermal emitter’s input power, as a function of time *t*; $C_e$ is the heat capacity of the thermal emitter; $T\left( t\right)$ is the transient temperature that varies between the lower extreme limit of the ambient temperature $T_0$ and its steady-state temperature; $k_e$ is the thermal conductivity; $\sigma _e$ is the Stefan–Boltzmann constant; $\varepsilon$ is the emissivity of the emitter; and $A_e$ is the heated surface area of the thin-film thermal emitter.

Calibrating the values of the relevant parameters $W_R$, $C_e$ and $k_e$ is critical for both cognitive synchronization and demodulation. In our recent work^8^, these values were simply estimated from typical specifications given by the manufacturer and, thus, did not take account of variations due to batch-to-batch manufacturing, component aging and operational environmental changes. In this section, we introduce an emitter calibration routine based on a zero-order optimization method that does not require changes in hardware.

Assuming the channel filters, atmospheric attenuation and pyroelectric sensor are all adequately characterized, the mean-absolute-error (MAE) between the expected (predicted by the model) and received signals, corresponding to a predefined training sequence ($v_{train}\left[ t\right]$ and $v\left[ t\right]$, respectively), is a non-analytical function of the emitter parameter values:

19 $$\begin{aligned} f\left( W_R,\ C_e,\ k_e\right) =\frac{1}{N}\sum _{t=1}^{N}{|v_{train}\left[ t\right] (W_R,\ C_e,\ k_e)-v\left[ t\right] |} \end{aligned}$$

Minimizing this error function gives adaptive emitter parameter values, making the prediction more consistent with the measurement. However, we can only evaluate $f\left( W_R,\ C_e,\ k_e\right)$ at a given point $x_0\doteq \left( W_{R0},\ C_{e0},\ k_{e0}\right)$ without any *a prior* knowledge of its gradient $\nabla _xf\left( x_0\right)$ - comprised of partial derivatives with respect to the individual emitter parameters. Therefore, to optimize this function without using the finite-difference method (which does not scale-up very well), a zero-order approach is adopted in which the gradient $\nabla _xf\left( x_0\right)$ and Hessian matrix $\nabla _x^2f\left( x_0\right)$ are estimated by sampling. The former can be achieved by evaluating the function values at $x_0$ with added noise vectors $\xi$ sampled from a diagonal multivariate Gaussian distribution; this method is known as evolution strategy^17^ in computer science literature. Applying a second-order Taylor expansion on *f*(*x*) at $x_0$ gives:

20 $$\begin{aligned} f\left( x_0+\xi \right) =f\left( x_0\right) +\ \xi ^T\nabla _xf\left( x_0\right) +\frac{1}{2}\xi ^T\nabla _x^2f\left( x_0\right) \xi +O(\xi ^3) \end{aligned}$$

where $\xi ^T$ is the transpose of $\xi$. When sampling $\xi$ from $N(0,\ \sigma ^2I)$ - being the diagonal multivariate Gaussian distribution with zero-mean and $\sigma ^2I$ being the covariance matrix—we neglect the higher-order terms $O(\xi ^3)$, multiple both sides of Eq. (20) by $\xi$ and take the expectation to give:

21 $$\begin{aligned} \mathbb {E}_{\xi \sim N\left( 0,\ \sigma ^2I\right) }\left[ \xi f\left( x_0+\xi \right) \right] \approx \mathbb {E}_{\xi \sim N\left( 0,\ \sigma ^2I\right) }\left[ \xi f\left( x_0\right) +\ {\xi \xi }^T\nabla _xf\left( x_0\right) +\frac{1}{2}{\xi \xi }^T\nabla _x^2f\left( x_0\right) \xi \right] =\ \sigma ^2\nabla _xf\left( x_0\right) \end{aligned}$$

A Monte Carlo estimator for the gradient can then be constructed by drawing *K* I.I.D. samples from $N(0,\ \sigma ^2I)$:

22 $$\begin{aligned} \nabla _xf\left( x_0\right) = \frac{1}{K\sigma ^2}\sum _{k=1}^{K}{\xi _kf\left( x_0+\xi _k\right) } \end{aligned}$$

With knowledge of this gradient, any first-order optimization methods (e.g., gradient descent) can be used to minimize the error function from Eq. (19). To take advantage of second-order optimization methods (e.g., Newton-Raphson), having faster convergence, this sampling approach can be extended to Hessian matrix estimation^18^ as following:

23 $$\begin{aligned} \nabla _x^2f\left( x_0\right) \approx \frac{1}{K\sigma ^2}\sum _{k=1}^{K}{\frac{1}{\sigma ^2}f\left( x_0+\xi _k\right) \xi _k\xi _k^T-f\left( x_0+\xi _k\right) I} \end{aligned}$$

The emitter parameter values estimated in our recent work^8^ are used as initial values when solving the optimization problem. To ensure a physically meaningful value, an appropriate box constraint is applied to each emitter parameter. A small diagonal term may be added to the estimated Hessian matrix, to make it positive definite.

**Figure 3:**
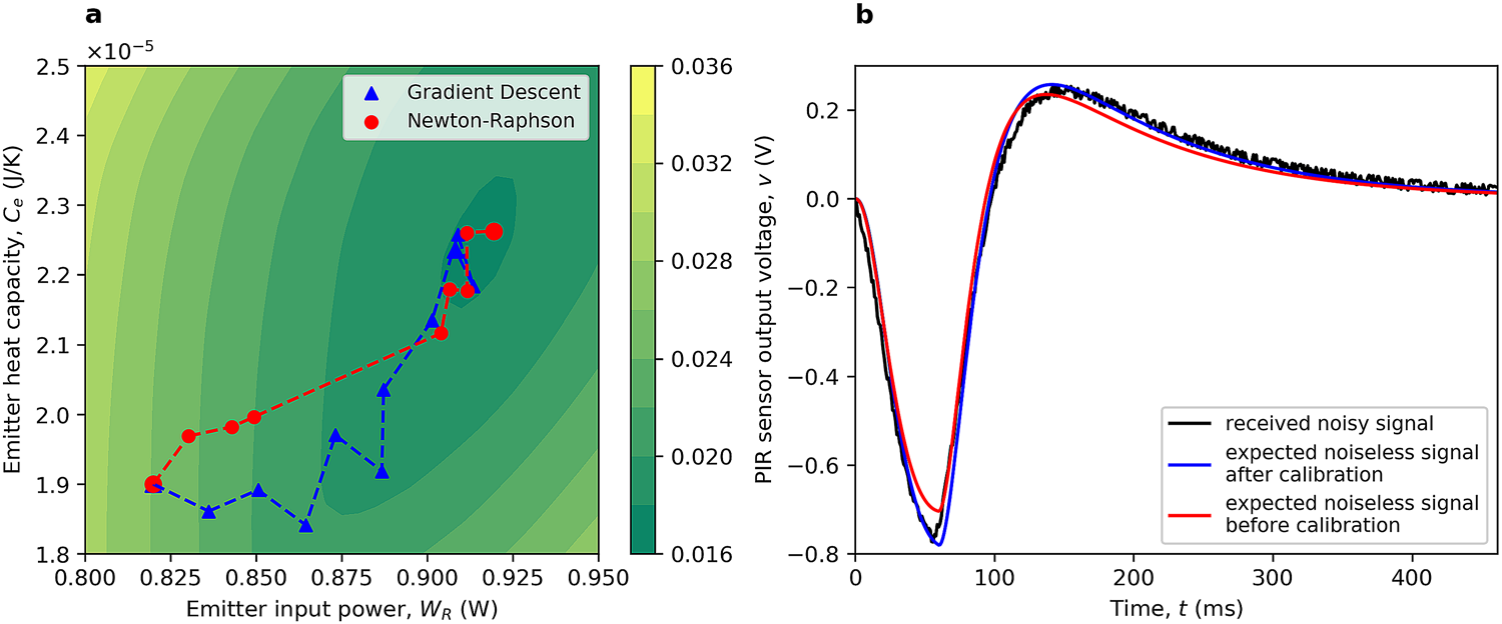
Thermal emitter calibration demonstrated for the 2 m single-channel ‘THz Torch’ wireless link field trial: (**a**) Contour plot of error function in Eq. (19) that depends on $W_R$ and $C_e$ ($k_e$ is fixed to $1.03\times {10}^{-3}$ W/K for visualization purposes). Optimization progress of the gradient descent and Newton-Raphson methods are indicated by the triangular and circle markers, respectively. (**b**) Synchronized received noisy signal with two expected noiseless signals that adopt the initial and optimized emitter parameter values.

Figure 3 shows the result of introducing the thermal emitter calibration technique. It can be seen from the contour plot of the error function in Eq. (19), shown in Fig. 3a, that the evolution of the optimization processes converge into the minimum area of this error function. In addition, the Newton-Raphson algorithm demonstrates a faster convergence rate by exploiting second-order derivatives. As shown in Fig. 3b, the expected noiseless signal with optimized emitter parameter values gives a better match with the received noisy signal. This clearly demonstrates the adaptive nature of our calibration approach.

### Synchronization under parameter mismatch

Timing synchronization locates the temporal boundary for each received signal by searching for the optimal time delay $\delta$. Poor synchronization misaligns the received signal to the transmitted symbol sequence and degrades the performance of calibration and demodulation. Two synchronization algorithms, using naive template matching and the matched filter, were introduced in the previous section. Both are based on template matching and assume the expected noiseless signal $v_{train}[t]$ corresponding to a predefined training sequence is accurate. However, producing an accurate expected noiseless signal relies on the calibrated thermal emitter and PIR sensor parameters. The initial emitter and sensor calibration sequences have to be synchronized using inaccurate expected noiseless signals. In this subsection, a change-point detection approach^19^ is exploited to improve timing synchronization for this initial scenario.

Change-point detection of a signal aims at finding some key timestamps at which a pre-defined pattern of the signal shows an abrupt change. This can be formulated as an optimization problem:

24 $$\begin{aligned} \min _\mathscr {I} \ \sum _{k=0}^{K-1} c(v[\mathscr {I}_k:\mathscr {I}_{k+1}]) \end{aligned}$$

where $\mathscr {I}$ is a set of ordered timestamps $\left\{ \mathscr {I}_k\right\} _{k=0}^K$, representing a specific way of segmentation; *v* is the complete discrete signal of length $T_H$; while $v[\mathscr {I}_k:\mathscr {I}_{k+1}]$ is one of the segments under $\mathscr {I}$ that includes $v[\mathscr {I}_k]$ and excludes $v[\mathscr {I}_{k+1}]$. For conciseness, $\mathscr {I}_0$ and $\mathscr {I}_K$ are fixed to 0 and $T_H$, respectively. Also, $c(\cdot )$ is a cost function that defines the changing pattern to be detected. Here, the number of change points $|\mathscr {I}|$ is set to a constant and the cost function is chosen to be:

25 $$\begin{aligned} c(v[\mathscr {I}_k:\mathscr {I}_{k+1}])=\min _{\alpha , \beta } \sum _{t=\mathscr {I}_k}^{\mathscr {I}_{k+1}-1} (v[t]-\beta -\alpha t)^2 \end{aligned}$$

This means that a piece-wise linear model, with a fixed number of knots, is fitted to the received signal and these knots are assigned to be the change points.

The improved timing synchronization now includes two phases: the template-matching phase, which achieves coarse synchronization due to the inaccurate expected noiseless signal corresponding to the transmitted training sequence; and the change-point detection phase. With the latter, the optimization problem in Eq. (24) is solved by dynamic programming, to obtain a set of change points $\left\{ \mathscr {I}_k^v\right\} _{k=0}^K$ from the received noisy signal pre-synchronized by the template-matching phase. The revised time delay can be simply decided by $\delta _{cp} = \delta _{tm} + \mathscr {I}_1^v$, where the index of the first change-point (as a correction) is added to the coarse time delay $\delta _{tm}$ from the template-matching phase.

The change-point detection phase is time-consuming and, thus, only used for the initial emitter and sensor calibrations. With the initially calibrated parameters, the expected noiseless signal will be accurate enough for simple template-matching based timing synchronization.

**Figure 4:**
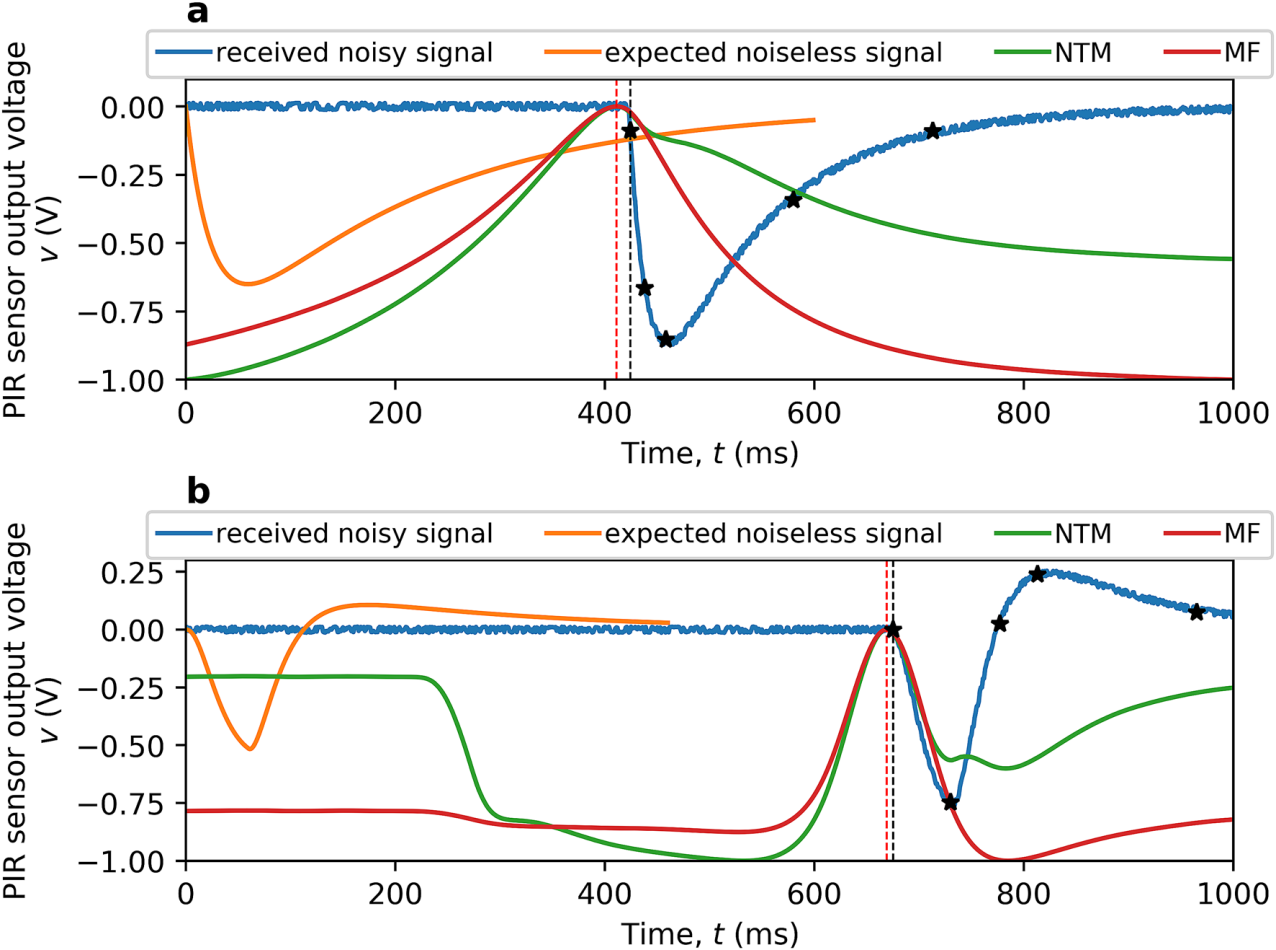
Improved timing synchronization using change-point detection demonstrated on the 2 m single-channel ‘THz Torch’ wireless link field trial. Compared to Fig. 2c,d, the expected noiseless signal (in orange) is produced by mismatched emitter and sensor parameters. The coarse time delay $\delta _{tm}$ decided by the template-matching phase is indicated (red dashed line). Black stars represent the detected five change points $\{\mathscr {I}_k^v\}_{k=1}^5$ and the black dashed line indicates the improved timing synchronization $\delta _{cp}$ after the change-point detection phase. (**a**) Timing synchronization for initial PIR sensor calibration. (**b**) Timing synchronization for initial thermal emitter calibration.

Figure 4 illustrates the operation with the improved timing synchronization. The emitter and sensor parameters are reset to their initial values when predicting the expected noiseless signal. Due to parameter mismatch, the time delay $\delta _{tm}$ given by template-matching based algorithms deviate from the optimal $\delta ^*$ shown in Fig. 2c&d. The deviation is then corrected by the change-point detection phase. In Fig. 4a, $\delta _{tm}=411$ ms, $\delta _{cp}=424$ ms and $\delta ^*=422$ ms. In Fig. 4b, $\delta _{tm}=669$ ms and $\delta _{cp}=\delta ^*=675$ ms. This shows that change-point detection is capable of achieving ideal timing synchronization under parameter mismatch.

### Introduction of neural network

Combined with thermodynamics-based modelling, template-matching based algorithms (NTM and MF) are capable of significantly improving the operational figure of merit (Range $\times$ Bit Rate) for ‘THz Torch’ wireless communications links. However, these solutions have a number of shortcomings:

- As given in Eq. (1), the received signal *v*[*t*] is distorted by the noise term $v_n[t]$. Due to unmodelled behaviour (e.g., environmental noise) and system parameter mismatch (e.g., calibration error), the equivalent $v_n[t]$ may not closely follow the WGN assumption, which ultimately degrades the bit-error-rate performance.
- When predicting expected noiseless signals $v_{S0}^{s_{1:(i-1)}}$ and $v_{S1}^{s_{1:(i-1)}}$, for demodulating the *i*-th symbol $s_i$, initial conditions are decided by the demodulation results for its previous symbols $s_{1:(i-1)}$. An error in $s_{1:(i-1)}$ will give the wrong initial condition, which may cause a chain of errors within the sequence.
- When numerically solving differential equations and integrals, there is a trade-off between computational complexity and accuracy. Although real-time communications is achieved, with low bit rates, a more lightweight algorithm is preferred; making it possible to further ease computational hardware requirements.

**Figure 5:**
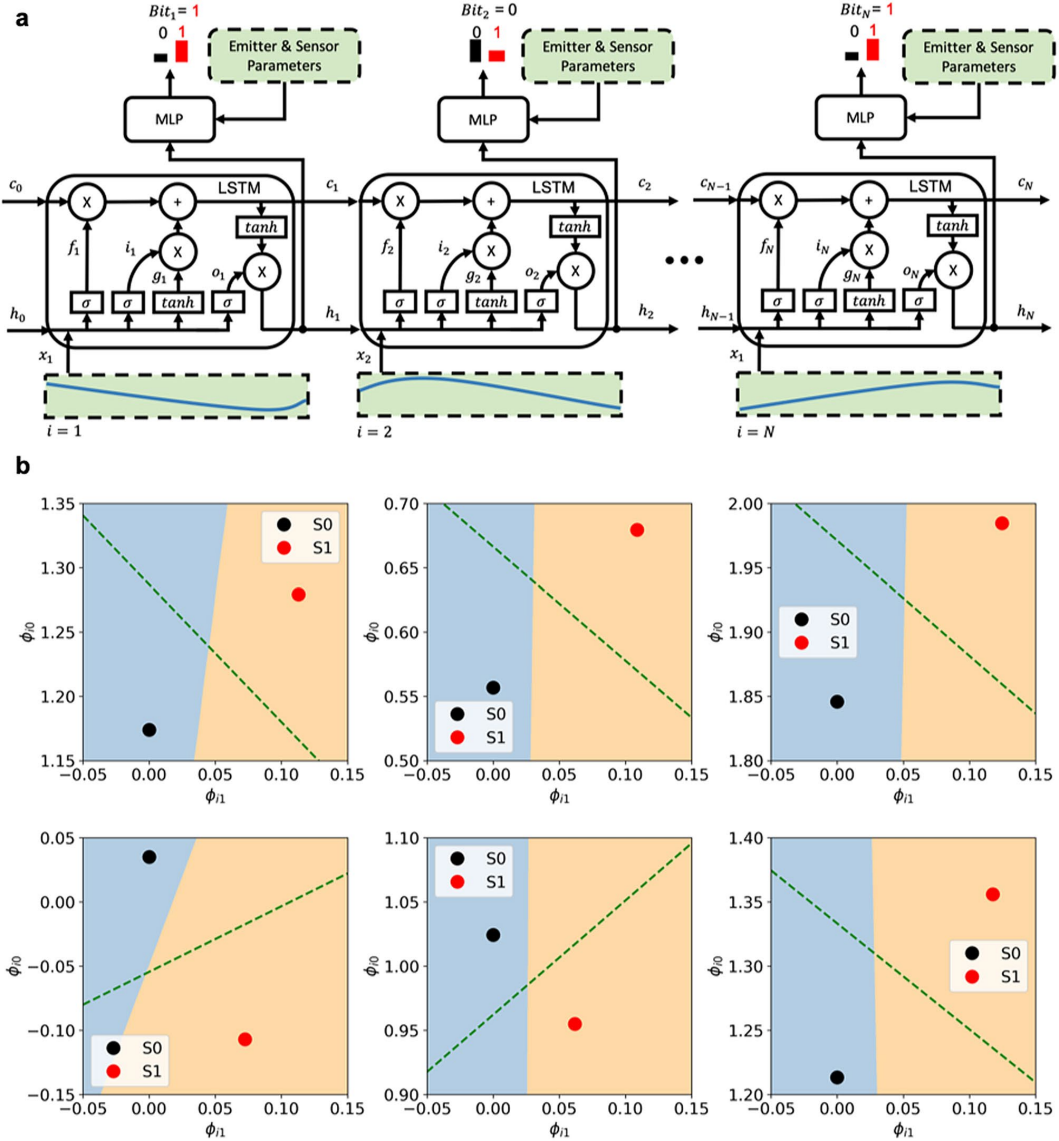
(**a**) Unrolled computational graph for the complete LSTM-based cognitive demodulator with emitter and sensor parameter inputs and received noisy signal (cyan curve) input across a sequence of *N* symbols. (**b**) Signal space decision boundaries for the LSTM-based demodulator (between the two colours) and the matched filter (green dashed line, mid-perpendicular of the line segment through S0 and S1) for six arbitrary sampled symbols.

To address all these shortcomings, we introduce a neural network (NN) based demodulator, which combines thermodynamics-based modelling with artificial intelligence. Unlike template-matching based algorithms, which employ a MLE rule, we exploit a Maximum A Posterior (MAP) rule. This directly approximates the posterior probability $p(s_i=S1|v)$ using a neural network. Here, a neural network (NN) is essentially an expressive function approximator composed of manually designed, parameterized, non-linear functions (layers). Since the NN is fully differentiable, the parameters can be learnt by the backpropagation algorithm, which is an application of the ‘chain rule’ in multivariable calculus. The recurrent neural network (RNN)^20^ is a family of neural networks having a special structure that is tailored for time series analysis. Inputs at all time steps (waveforms associated with bits in our case) are processed by a shared network, while a concise representation of the history is extracted and passed forward in time. Here, the Long-Short Term Memory (LSTM)^21^, as a standard RNN architecture, is exploited. The complete LSTM-based Cognitive Demodulator is illustrated in Fig. 5a. Expressions for calculating the intermediate variables in Fig. 5a are:

26 $$\begin{aligned} \begin{aligned} f_i&=Sigmoid\left( W_f\left[ x_i,\ h_{i-1}\right] +b_f\right) \\ i_i&=Sigmoid\left( W_i\left[ x_i,\ h_{i-1}\right] +b_i\right) \\ g_i&=tanh\left( W_g\left[ x_i,\ h_{i-1}\right] +b_g\right) \\ o_i&=Sigmoid\left( W_o\left[ x_i,\ h_{i-1}\right] +b_o\right) \\ c_i&=f_i\otimes c_{i-1}+i_i\otimes g_i \\ h_i&=o_i\otimes {tanh(c}_{i-1}) \end{aligned} \end{aligned}$$

where *i* is used to index the waveform $x_{i}$, associated with the *i-*th bit; $W_{f,i,g,o}$ and $b_{f,i,g,o}$ are internal parameters for the LSTM; $\left[ x_i,\ h_{i-1}\right]$ represents the concatenation of $x_i$ and $h_{i-1}$; $Sigmoid\left( x\right) =\left( 1+e^{-x}\right) ^{-1}$ is the sigmoid function; and $\otimes$ represents the Hadamard product. For each bit, the LSTM takes the synchronized measured waveform associated with this bit to update its cell state $c_i$ and hidden state $h_i$ from their previous states. While the updated states are passed to the next step, hidden state $h_i$ is concatenated with emitter and sensor parameters. The resulting vector, which includes both the features of the system and the representation of the measured waveform, is forwarded to a Multi-Layer Perception (MLP) to make a probabilistic decision for the current bit.

Two issues that prevent a direct application of LSTM-based Cognitive Demodulator are overfitting^22^ and domain-shift^23^. While LSTM has the potential to approximate sophisticated mapping from measured waveforms into binary bits, it may be overfitted to the collected training dataset (instead of capturing the real underlying mapping). The solution is to collect a large training dataset, but this may be time-consuming in a real system. Domain-shift occurs in the LSTM-based Cognitive Demodulator, as the ‘THz Torch’ wireless links have time-variant emitter and sensor parameter values. A conventional learning-based demodulator may work poorly on the system with parameter values that are not covered by its training dataset. However, the complete thermodynamics-based model, introduced in the previous section, can be employed to solve both the overfitting and domain-shift issues, by building a training dataset through simulations.

The Cognitive Demodulator is trained on this simulated training dataset by minimizing the well-known binary cross-entropy loss function:

27 $$\begin{aligned} Loss=\ -\sum _{n=1}^{N}\sum _{m=1}^{M}{s_{n,m} \ ln\ {\left( {\hat{s}}_{n,m}\right) }+\left( 1-s_{n,m}\right) \ ln\ {\left( 1-{\hat{s}}_{n,m}\right) }} \end{aligned}$$

where *N* is the number of frames in the training dataset; *M* is the number of bits per frame; $s_{n,m}$ is the ground-truth binary value of the *m*-th bit in the *n*-th frame, while ${\hat{s}}_{n,m}$ is the corresponding predicted probability of being binary one. Finally, the LSTM-based Demodulator can be directly deployed to the real ‘THz Torch’ wireless link. The internal parameters for the LSTM and MLP can be further fine-tuned by using measured waveforms and also emitter and sensor parameter values from the real system (known as transfer learning^23^).

To understand the underlying decision-making process, operated by the trained LSTM-based demodulator, Fig. 5b shows the decision boundaries for six arbitrarily sampled received symbols and makes a comparison with those from the matched filter. By inspection of the decision boundaries in Fig. 5b , it can be seen that the matched filter is always optimal with purely WGN. However, in practice, the system noise is not purely white Gaussian and it will be demonstrated in the next section that the LSTM-based demodulator gives a better performance when compared with the matched filter. Each decision boundary for the matched filter is obtained by equalling the probabilities of the received signal in Eq. (17) under two Gaussian distributions in Eq. (16), while the LSTM decision boundary is estimated numerically by computing $\hat{s}_i=p(s_i=S1|v_{s_i}^{s_{1:(i-1)}}[t])$ over a signal space grid. Being different from the matched filter boundary, which is always the mid-perpendicular of the line segment through *S*0 to *S*1, the local LSTM boundary resembles a straight line whose position and rotation varies from symbol to symbol. Another noticeable pattern is that the LSTM boundary tends to be parallel to the first basis $\phi _{i0}$ given in Eq. (15).

The LSTM-based Cognitive Demodulator overcomes the shortcomings of our recent work^8^, in several respects:

- The LSTM is independent of the emitter and sensor parameter values. The rationalé is that although these hardware parameter values may vary between systems, the measured waveforms have similar patterns for a particular sequence of bits. The LSTM will extract these meaningful patterns, instead of being distracted by variations in hardware parameter values. Therefore, demodulation can be less sensitive to calibration accuracy.
- Noise is taken into account by adding tailored artificial noise to the training dataset. This improves the demodulator’s resilience to noise.
- Demodulation of the current bit is not directly dependent on the correct result of the previous bit. Instead, the whole measured waveform within the complete frame is used; eliminating the possibility for a chain of errors.
- The computational cost is significantly reduced, to a simple forward pass of the NN.

Although artificial intelligence is exploited, demodulation remains rooted in the thermodynamics-based model. When making the final decision, the demodulator still takes the hardware parameter values into consideration, via the MLP. Furthermore, training is based on simulated data from the thermodynamics-based model. In another word, the Cognitive Demodulator process is distilled into the lightweight NN.

### Enhanced performance demonstration

**Figure 6:**
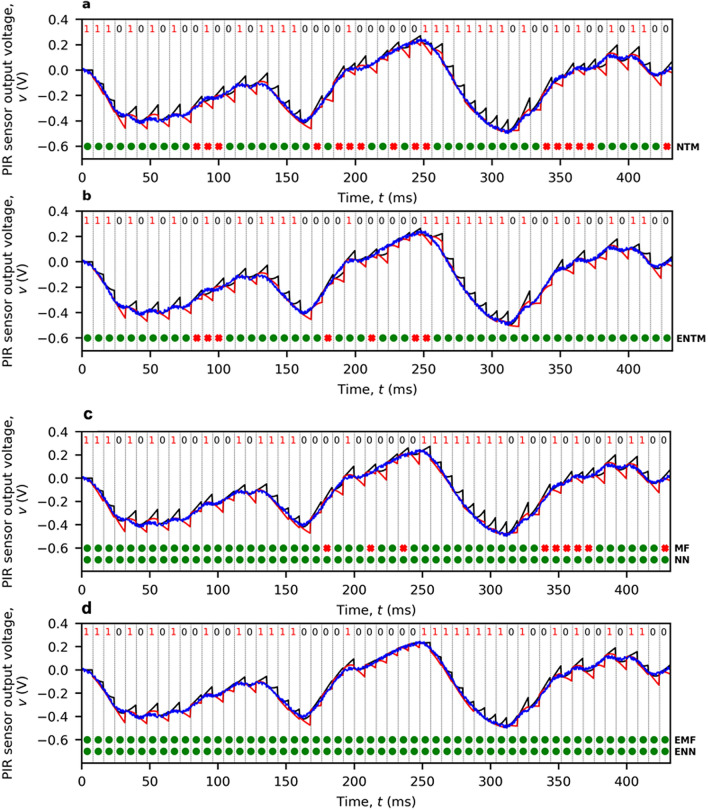
Operation of demodulation demonstrated for the 2 m single-channel ‘THz Torch’ wireless link field trial. (**a**) NTM with initial thermal emitter parameters (16 symbol errors). (**b**) NTM with calibrated thermal emitter parameters (7 symbol errors). (**c**) MF with initial thermal emitter parameters (9 symbols errors). (**d**) MF with calibrated thermal emitter parameters (error free). The LSTM-based demodulator with and without calibrated thermal emitter parameters (error free) is also shown in (**c**,**d**). Here, the LSTM-based demodulator is only trained on the simulated dataset without any fine-tuning using data from the real system.

Figure 6 show the operation of demodulation with three different algorithms. All the figures use the same test frame, which is collected from the single-channel 2 m link field trial, transmitting at 125 bps and composed of a four-symbol preamble followed by 50 pseudo-randomly generated symbols. The received noisy signal *v*[*t*] is shown in blue. For the naive template matching and matched filter algorithms in Fig. 6, two expected noiseless signals for each symbol $s_i$ ($v_{S0}^{s_{1:(i-1)}}$ and $v_{S1}^{s_{1:(i-1)}}$) are shown in black and red, respectively. In each figure, the correctness of the demodulated symbols is indicated by a sequence of green circles and red crosses. To highlight the contribution from the calibrator, we also compare two configurations for each algorithm. The configuration with calibrated thermal emitter parameters is labelled with a prefix ‘E’ and that without the prefix employs initial emitter parameters.

An algorithm-level comparison reveals that the matched filter consistently outperforms our previously reported naive template matching^8^. This is achieved by employing the signal space concept to eliminate any noise component that is orthogonal to the two-dimensional signal space. In contrast, with LSTM-based demodulation, there is no need to dynamically produce the expected noiseless signals; This has an ideal performance, resulting in error-free transmission for this particular test frame. In addition, both template-matching based algorithms (NTM and MF) show a chain of symbols with errors. As explained earlier, this pattern is caused by the dependency of the currently expected signals on previously demodulated symbols $s_{1:(i-1)}$. Moreover, calibrating the emitter parameters can significantly enhance both template-matching based algorithms by providing more accurate ‘templates’. In contrast, with LSTM-based demodulation, good resilience is shown to parameter mismatch in this test scenario. This is because the LSTM network is trained to extract common signal patterns shared between different parameters. Bit error rate (BER) measurements will show that combining the calibrated parameters through the MLP layers enhances the performance of the LSTM-based demodulator.

**Figure 7:**
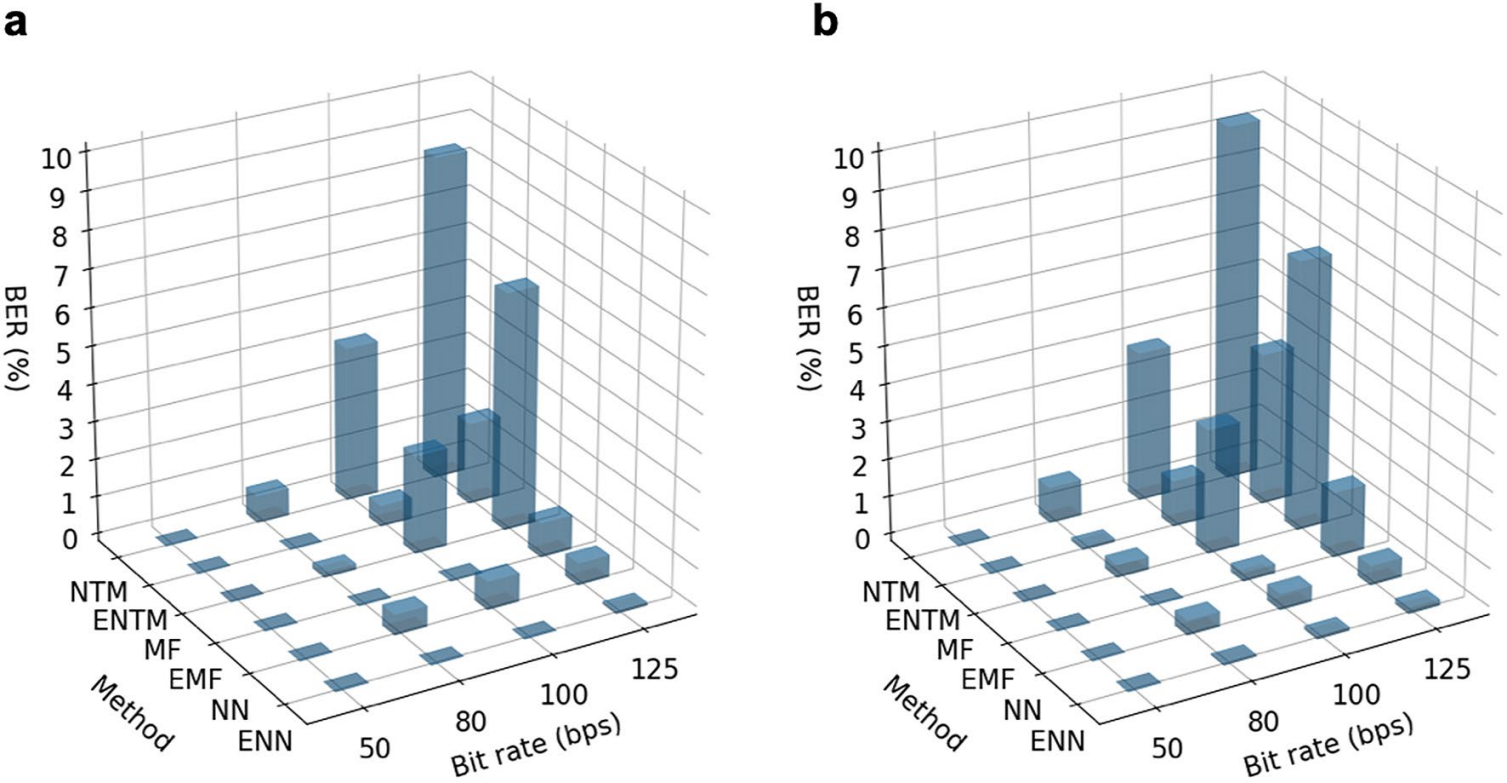
Measured BER performance for the single-channel 2 m link. (**a**) in a laboratory (reference) environment with optical bench and precision alignment rails^8^. (**b**) in a non-laboratory (real-world) environment (office corridor) with laser alignment, as shown in Fig. 1a–d.

Figure 7 show the measured raw BER performances (without any additional error correction coding) for the single-channel 2 m wireless links in both the laboratory and non-laboratory environments. These measurements are performed by sending 2000 frames across the wireless link; each frame has a fixed four-symbol preamble for timing synchronization and 50 pseudo-randomly generated symbols. The fractionally spaced sampler is set to 20 times the baud rate. The discrete PIR sensor output voltage signal for each frame is saved and then demodulated offline, using our six algorithms for comparison: (E)NTM, (E)MF, (E)NN. Again, with LSTM-based demodulation, (E)NN are only trained on the simulated dataset, without any fine-tuning using data from the real system.

As a baseline, the NTM algorithm gives a BER of less then 9% at 125 bps. The ENTM algorithm maintains the BER to below 5%, which is still impractical for most applications. When operating at a low symbol rate, the EMF algorithm, enhanced by the thermal emitter calibration, shows a significantly improved performance. Introducing the neural network provides a step-change reduction in the BER, down to 1%; even with mismatched emitter parameter values. Finally, the ENN algorithm that combines all enhancement techniques consistently outperforms the other algorithms at higher bit rates, with measured BER less than 0.16% at 125 bps. When compared to our NTM baseline, its BER is improved by a factor of 108 within the laboratory environment and 59 in the non-laboratory field trials. An additional non-laboratory experiment was performed over an extended 2.5 m link, which demonstrated error-free transmission of data at 50 bps. Moreover, with a 1.5 m link, the ENN algorithm demonstrated a measured BER below 0.1% at 125 bps.

From the measured results, the following three conclusions are:

- With the non-laboratory demonstrators, the performance is slightly degraded due to sub-optimal spatial alignment and possibly more environmental noise.
- From the algorithm-level comparison, the match filter consistently outperforms naive template matching, while LSTM-based demodulation achieves best performance in all test scenarios.
- Emitter parameter calibration can enhance all three algorithms. The two template-matching based algorithms (NTM and MF) heavily rely on accurate parameters, while LSTM-based demodulation is more resilient to parameter mismatch.

From a batch test of 100 frames on a Intel Xeon 2.30 GHz CPU, with (E)NTM and (E)MF, the time taken for demodulation is approximately 2.54 seconds per frame; while with (E)NN the time taken is only 0.256 milliseconds per frame. Therefore, introducing the neural network speeds-up demodulation by a factor of 9,922; allowing for real-time demodulation, while easing computational hardware requirements.

## Discussion

In this work, several enhancements for cognitive demodulation are presented. By calibrating the thermal emitter parameter values, using a zero-order optimization approach, the demodulation algorithm becomes more adaptive to component variations and environmental changes. Being an asynchronous wireless link, the timing synchronization technique that exploits change-point detection achieves a better temporal alignment between the measured waveform and its associated frame. Finally, an LSTM-based demodulator is introduced, which significantly improves both the BER performance and computational efficiency.

This paper demonstrates a clear paradigm shift in the way BER can be reduced for the generic ‘THz Torch’ technology with software advances. Future work will focus on returning to improvements in hardware, to advance the operational figure of merit. For example, the miniature thermal emitter can be replaced by the more powerful Globar^24^, to increase signal power (albeit with a longer thermal time constant), which will increase both Range and Bit Rate.

System noise has various sources^4^: internal (e.g., from the PIR sensor, which includes the built-in transimpedance amplifier and external unity-gain voltage-follower) and environmental (e.g., electromagnetic interference, e.g., 50 Hz interference from mains power supply picked-up^8^; microphonic noise; and low frequency ambient temperature fluctuations^8^).

**Figure 8:**
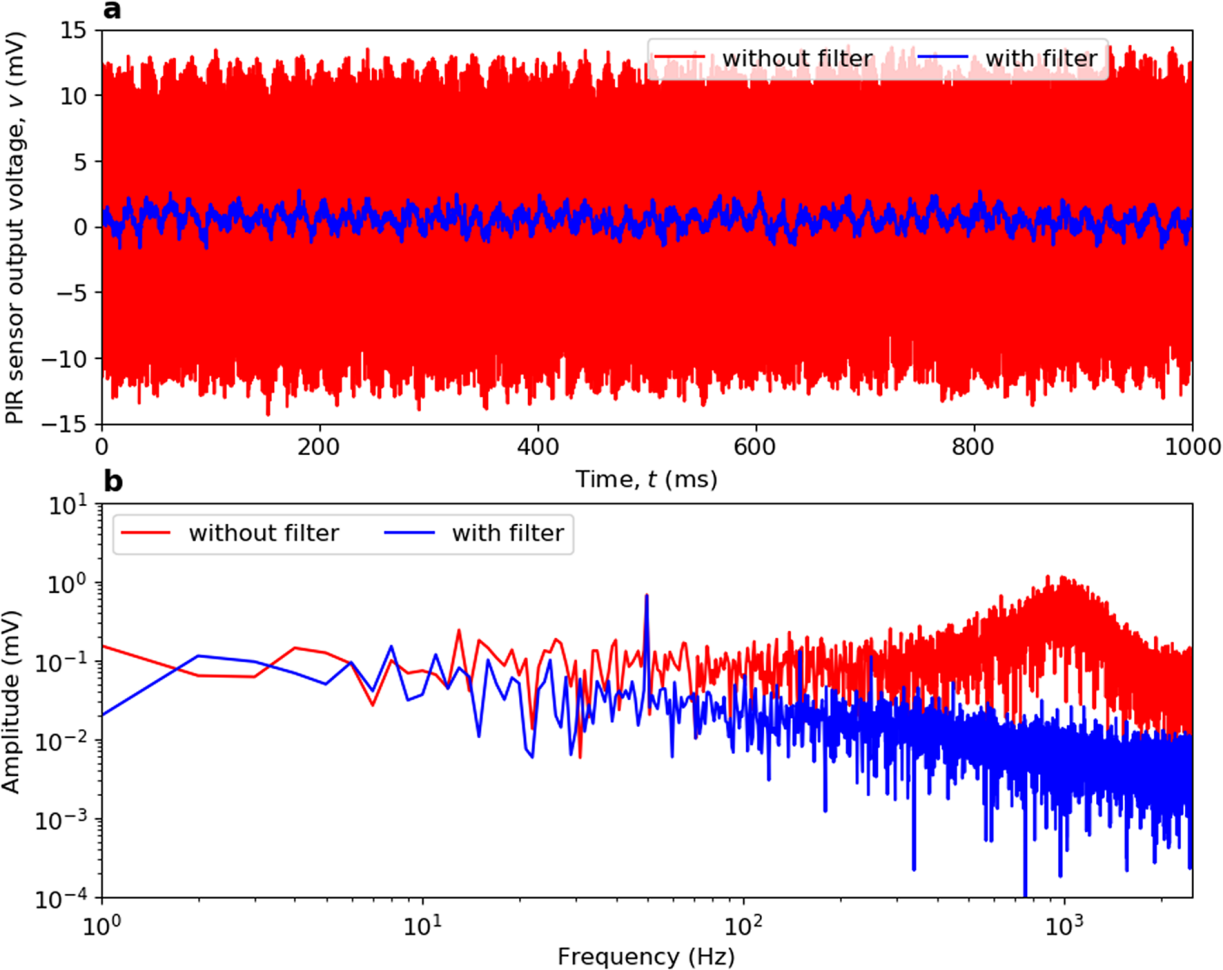
Measured background noise within the laboratory environment with PIR sensor shrouded. (**a**) output noise voltage. (**b**) associated single-sided amplitude spectral responses. The red plots represent unfiltered measurements and the blue plots represents the addition of an anti-aliasing filter, inserted between the PIR sensor and ADC, having a cut-off frequency of 2.5 kHz.

Assuming a noiseless PIR sensor, an anti-aliasing filter is not required at the ADC input. The reason is that our PIR sensor has peak responsivity at $\sim$3 Hz and a 20 dB/decade frequency roll-off characteristic. As a result, with transmission at 125 bps there is no significant frequency component at half the 2.5 kHz sampling frequency. However, given the broad frequency spectrum of our system noise, the introduction of a band-limiting filter would provide a significant enhancement to the signal-to-noise power ratio performance. In turn, both Range and Bit Rate can be increased, and could even facilitate *M*-ary amplitude shift keying signalling. For example, a simple anti-aliasing low-pass filter, having a 2.5 kHz cut-off frequency, was introduced for a sampling frequency of 5 kHz. The background noise was measured up to the Nyquist frequency, shown in Fig. 8, with and without the filter. From Fig. 8a, it can be seen that there is a factor of 10.02 reduction in RMS noise voltage, corresponding to a 20 dB reduction in noise power (i.e., resulting in the same enhancement of signal-to-noise power ratio). In practice, the anti-aliasing low-pass filter should be based on a Bessel approximation, since a constant differential-phase group delay is required within the signaling passband to maintain the shape of the measured waveform.

The environment noise, which tends to dominant system performance, requires further investigation. For example, in Fig. 8b, electromagnetic interference can be seen at 50 Hz. With microphonic noise, anti-vibration material can be employed to package and mount the PIR sensor. The introduction of a 3D-printed shroud has already demonstrated a significant reduction in noise related to both microphonic and ambient temperature fluctuations. While a great deal of care went into shielding against relatively low frequency electromagnetic interference (and decoupling intrinsic electronic noise), there is more scope for further investigation.

Cognitive Demodulation (in its various forms) has been applied to thermal-based emitter and sensor front-end hardware. However, the concept can potentially be adapted to any transmit-receive technologies, where sufficient sampling can be performed within the transient time of signalling. Enhancing operational speeds in this way could open-up many new applications for future communications and sensor technologies. With the later, our engineered thermal source can be replaced by bodies that naturally emit thermal radiation; monitoring for specific thermal transients can find low-cost applications in non-destructive testing and remote sensing. For example, analysing temporal fluctuations in localized body temperature could provide useful information in medical diagnostics; while within the aerospace sector, sensor arrays could be deployed for monitoring anomalous behaviour at a distance. An extreme example is with broadband (sub-)millimeter-wave astronomy, traditionally employing microbolometer arrays, where neural networks can be trained to detect the very early onset of astronomical events that can be modelled. Moreover, with room-temperature terahertz and thermal infrared cameras, which employ microbolometer arrays, the thermal time constant constraints of the detectors can be overcome by adapting the techniques presented here to significantly increase frame rates.

## Methods

### Thermal emitter

The typical DC power consumption of the thermal emitter is $W_R$ = 134 mA $\times$ 6.7 V = 898 mW. With a pseudo-random binary sequence of transmit bits, the time-average power consumed by the 1.7 mm $\times$ 1.7 mm = 2.89 mm$^2$ thermal emitter is only approximately 450 mW. Emitter calibration is achieved by sending a standard data frame through the wireless link and extracting the measured waveform associated with the frame header (preamble). The calibration procedure then optimizes the emitter parameter values to minimize the mean-absolute-error between the predicted and measured waveforms.

### Analog to digital converter (ADC)

In our most recent work^8^, the ADC used was the 13-bits NI USB-6009, giving a theoretical dynamic range of 80 dB. In this work, the 16-bit NI USB-6363 is used, giving an improved dynamic range of 96 dB. Moreover, previously^8^, the sampling frequency was fixed at 2 kHz for all bit rates; while here the sampling frequency is adapted to 20 $\times$ bit rate.

### Neural network

Given a frame containing a 4-bit preamble and 50 pseudo-randomly generated bits, the associated PIR sensor output voltage waveform can be simulated using our thermodynamics-based model, having random values for the emitter and sensor parameters. A NN training sample consists of the frame and its corresponding simulated waveform; 10,000 of these training samples make up the complete NN training dataset. This is followed by injecting additive white Gaussian noise into the simulated waveform (attempting to mimic measured waveforms from the PIR sensor). The LSTM-based demodulator is then trained on this dataset for a maximum of 20 epochs. To prevent overfitting to the training dataset, 20% of the training samples are reserved as a validation dataset, for early stopping. The ‘Adam’ optimizer^25^ is employed with a learning rate of $3\times {10}^{-4}$ and 100 samples per batch (with 100 batches per epoch in our case). Note that, for each training sample, the PIR sensor output voltage waveform could be measured from the ‘THz Torch’ wireless link. This would have the advantage of being more realistic (i.e., a real-world system, including both hardware and added noise contributions), however, collecting the training dataset would be more time consuming if random-valued emitter and sensor parameters are chosen for each frame.

### BER measurement

The raw BER is measured by sending 2000 frames across the wireless link; each frame has a ‘1110’ preamble and 50 pseudo-randomly generated bits. The received noisy signal for each frame is saved and then demodulated offline using our six algorithms for comparison: (E)NTM, (E)MF, (E)NN. The LSTM-demodulator used for BER measurements employs only the simulated training dataset, which provides greater efficiency.

### Noise measurement

For the noise measurements, there was a 20 s sampling period with a sampling frequency of 20 kHz in our recent work^8^; while here a 1 second sampling period is chosen with a sampling frequency of 5 kHz. A passive first-order Butterworth low-pass filter was introduced, inserted between the PIR sensor and ADC, consists of a series resistor (77 k$\Omega$) and shunt capacitor (820 pF), giving a -3 dB cut-off frequency of 2.5 kHz.
